# Aggressive angiomyxoma of the vagina: A case report and literature review

**DOI:** 10.1097/MD.0000000000041287

**Published:** 2025-01-24

**Authors:** Bo Ram Yu, Won Ku Choi, Dong Hyu Cho, Na-Ri Lee

**Affiliations:** a Department of Obstetrics and Gynecology, Jeonbuk National University Hospital, Jeonbuk National University Medical School, Jeonju, Republic of Korea; b Department of Internal Medicine, Division of Hematology and Oncology, Jeonbuk National University Hospital, Jeonbuk National University Medical School, Jeonju, Republic of Korea; c Research Institute of Clinical Medicine, Jeonbuk National University, Biomedical Research Institute of Jeonbuk National University Hospital, Jeonju, Republic of Korea.

**Keywords:** aggressive angiomyxoma, hormonal treatment, surgical treatment, vagina

## Abstract

**Rationale:**

Aggressive angiomyxoma (AAM) is an exceptionally rare mesenchymal tumor that predominantly manifests in the female genital organs during the reproductive age. Its rarity alone makes it a fascinating subject for study. The diagnosis of AAM necessitates differentiation from other benign or mesenchymal tumors and can be confirmed through immunohistochemistry (IHC) staining. Surgical resection is the primary treatment, and adjuvant treatment can be used as hormonal therapy with gonadotropin-releasing hormone agonists, selective estrogen receptor modulators, and aromatase inhibitors.

**Patient concerns:**

A 44-year-old premenopausal Korean woman presented with a growing perineal mass and frequent urination.

**Diagnoses:**

Histopathological findings confirmed AAM, with IHC staining showing estrogen receptor, progesterone receptor, actin and desmin positivity, and CD34 and S100 negativity.

**Interventions:**

The mass was excised transvaginally under general anesthesia.

**Outcomes:**

The patient showed no signs of recurrence 6 months postoperatively.

**Lessons:**

AAM in the vagina is a rare tumor that requires differential diagnosis using IHC staining. Previously, we reviewed reported cases and confirmed the feasibility and effectiveness of surgery as the main treatment. This might reassure us about the potential successful treatment of AAM. Adjuvant hormonal therapy with gonadotropin-releasing hormone agonists, selective estrogen receptor modulators, and aromatase inhibitors can further reduce the risk of recurrence.

## 1. Introduction

Aggressive angiomyxoma (AAM) is an exceedingly rare benign mesenchymal tumor, with most reported cases occurring in women. AAM commonly occurs in the pelvis and vulva of premenopausal women and is affected by high serum estrogen levels. Moreover, AAM has been found in other parts of the body, such as the testes and epididymis, although at a markedly lower frequency.^[1]^ The presence of estrogen receptor (ER) and progesterone receptor (PR) on the tumor surface has been confirmed in several case reports,^[2]^ which is considered a contributing factor to its occurrence, mainly during the reproductive years. However, because AAM occasionally occurs in adolescents and postmenopausal patients, sex hormones are not strictly essential for tumor development. Although it is a rare tumor, the incidence rate is reported to be high at approximately 60.1% in Asians, especially Chinese individuals.^[3]^ In South Korea, 6 cases were reported in 2014,^[4]^ with no additional reports thereafter.

Assuming AAM as the final diagnosis preoperatively or intraoperatively is challenging because of its rarity and need for pathological confirmation. Differential diagnoses should initially consider benign tumors, such as Bartholin gland cyst, Skene gland cyst, uterine fibroids, and other mesenchymal tumors. Misdiagnosis as a benign tumor is common; a definitive diagnosis is often made postoperatively through histopathological examination. If AAM is diagnosed via incisional biopsy preoperatively, surgical excision is the treatment of choice. However, it is important to note that local recurrence is possible even after surgical excision, which is why follow-up and hormonal therapy are crucial. These measures can reduce estrogen levels and recurrence rates, ensuring comprehensive postoperative care.

We present the case of a preoperative misdiagnosis of a posterior urethral mass, presumed to be a fibroid, which was confirmed as AAM postoperatively and review existing literature on AAM.

## 2. Case report

**Figure 1.**
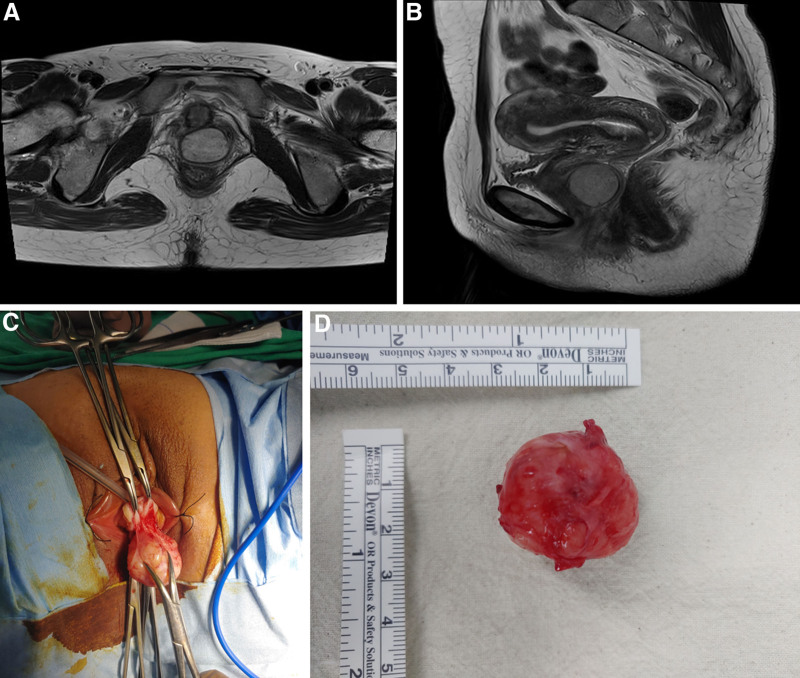
Pelvic magnetic resonance imaging (A) shows an approximately 2.5 cm round mass in the vagina on T2-weighted transverse image and (B) T2-weighted sagittal image. The mass was excised tranvaginally (C) and measured 3 × 3 cm in size grossly (D).

**Figure 2.**
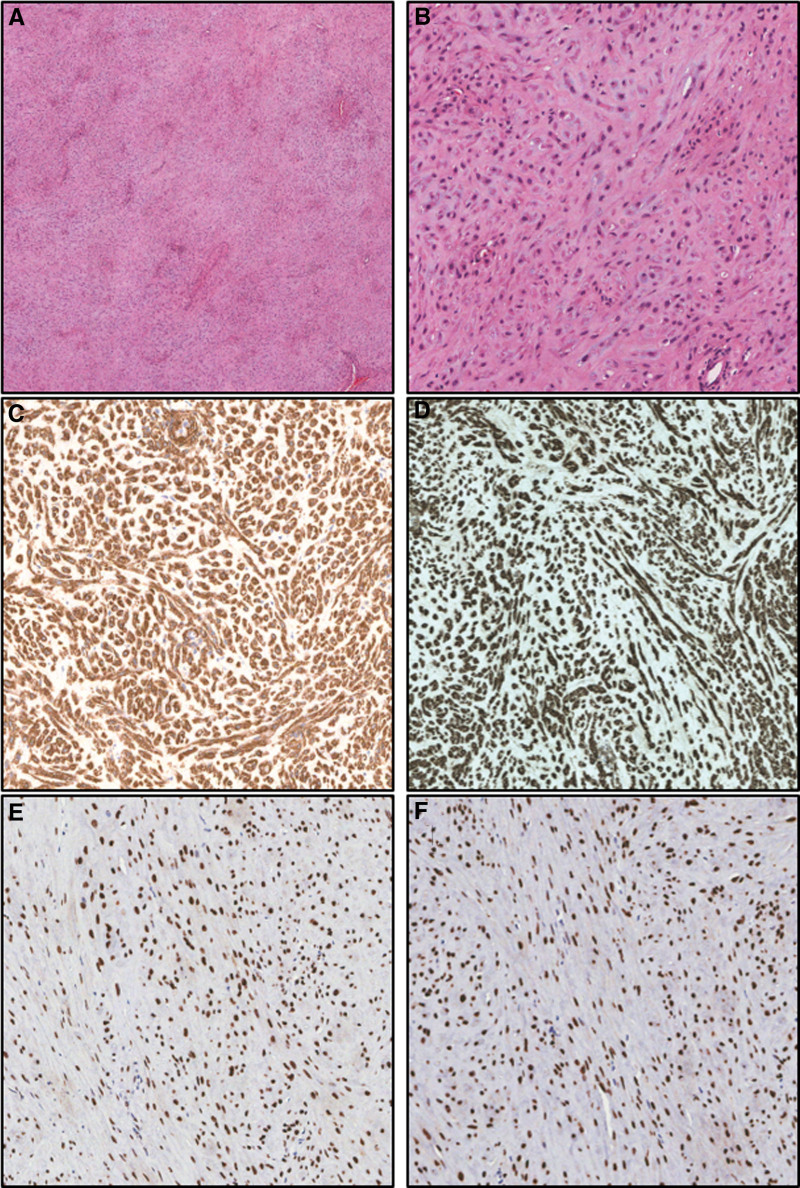
Histological examination of aggressive angiomyxoma. (A) Low magnification image of the tumor demonstrates the characteristic hypocellular stroma with prominent myxoid changes (H&E stain, original magnification: 40×). (B) High magnification image shows the intricate vascular network within the myxoid stroma (H&E stain, original magnification: 200×). Tumor cells show immunohistochemical expression for smooth muscle actin (C), desmin (D), estrogen receptor (E), and progesterone receptor (F) (original magnification: 200×).

A 44-year-old premenopausal woman presented with a growing perineal mass and frequent urination. The patient was not pregnant and had 2 live births (G2P2). The mass had been observed for 2 years, during which the patient experienced worsening urinary symptoms despite medication. On referral to our gynecology department, a tumor was identified beneath the urethra. The mass, approximately the size of a ping-pong ball, was pedunculated, located at the 12 o’clock position on the anterior vaginal wall, and was soft, non-fixed, and painless. Pelvic magnetic resonance imaging (MRI) revealed a 2.5 cm round tumor with high signal intensity on T2-weighted image, distinct from the surrounding organs (Fig. 1A and B). Parasitic fibroid or benign soft tissue tumor was diagnosed preoperatively, and the mass was excised transvaginally under general anesthesia (Fig. 1C). Grossly, the tumor was round, encapsulated, and 3 × 3 × 2.5 cm in size (Fig. 1D). Postoperative pathology confirmed AAM, with immunohistochemistry (IHC) staining showing actin and desmin positivity and CD34, cytokeratin, and S100 negativity (Fig. 2). Additionally, ER and PR were strongly positive, with a score of 8 (5 + 3 [proportion score + intensity score]) according to the Allred scoring system. The patient was discharged without complications on postoperative day 4 and remained symptom-free with no recurrence for 6 months postoperatively.

## 3. Discussion

Pathological confirmation, involving IHC staining of ER and PR, is required for the diagnosis of AAM. Besides ER and PR, other markers, such as CDK4, were tested; however, CDK4 shows weak responses and was less helpful for diagnosis. ER and PR showed 87.5% positivity but were not specific markers, as they reacted in other conditions.^[5]^ A recent immunohistochemical analysis of 87 patients reported ER and PR positivity rates of 97.4% and 91.9%, respectively, with vimentin observed in all patients,^[3]^ Recently, HMGA staining has been frequently observed in AA specimens (68%) and can be used as a diagnostic marker. HMGA staining was observed in other mesenchymal tumors, such as leiomyomatous neoplasms (44%) and fibroepithelial stromal polyps (19%).

Aside from HMGA2 staining, genetic testing for HMGA2 was performed. HMGA2 is located on the long arm of chromosome 12 (12q) and associated with translocation. Research has indicates that somatic mutations in HMGA2 may be linked to the pathogenesis of AAM.^[6,7]^ HMGA2-YAP1 gene translocation has been identified,^[8]^ and patients with HMGA2 gene mutation respond to estrogen antagonism.^[8,9]^ Although this suggests its potential use as a diagnostic marker and prognostic indicator, further studies are warranted. In this case, HMGA staining and next-generation sequencing-based HMGA2 mutation testing were not conducted owing to financial and methodological limitations.

Surgical excision is the primary treatment of AAM. For extensive tumors, potential functional impairments and cosmetic changes must be considered during resection. Thus, imaging studies, such as computed tomography and MRI, are needed to determine the surgical range preoperatively, confirming the three-dimensional structure of the tumor and its connection to surrounding organs. Presently, pelvic MRI revealed clear margins without organ involvement, allowing local excision. For large tumors with clear margins, a local skin graft may minimize deformity postoperatively.^[10,11]^ This patient had a relatively small, pedunculated tumor, allowing for complete local excision without functional or cosmetic concerns.

AAM rarely shows distant metastasis pre- and postoperatively, but has a relatively high local recurrence rate of 19.7% to 27% owing to surrounding tissue infiltration.^[3,12]^ There was no statistically significant difference in the recurrence rates, despite positive resection margins.^[12,13]^ To reduce recurrence, adjuvant treatments such as hormone therapy with gonadotropin-releasing hormone (GnRH) agonists, selective estrogen receptor modulators (SERMs), and aromatase inhibitors (AIs), may be considered. GnRH agonists are promising as adjuvant therapy to lower serum estrogen levels for positive resection margins and incompletely excised tumors.^[14]^ SERM monotherapy and combination therapy with GnRH agonists have been reported to be effective in similar situations.^[15–17]^ In postmenopausal women, letrozole, an AI, has been suggested to reduce peripheral estrogen production and prevent relapse.^[18,19]^

GnRH agonists inhibit growth and reduce the size of ER-positive tumors, as evidenced in several reports that mainly involved premenopausal women in whom GnRH agonists were used to reduce tumor size preoperatively^[10,20]^ or prevent progression postoperatively, with some cases achieving complete remission.^[2,21]^ From January 2011 to March 2024, leuprolide, triptorelin, and goserelin have been commonly used, with treatment durations ranging from 3 months to lifetime (Table 1).^[13,16,17,20,22–37]^ Most patients reported a 6-month treatment duration. Some case reports lacked detailed treatment duration and follow-up data, necessitating further detailed case studies to statistically compare treatment outcomes. Long-term GnRH agonist use can cause hypoestrogenic symptoms, such as vasomotor symptoms and osteoporosis; thus, requiring follow-up on patient tolerance, serum estrogen status, and bone mineral density, especially in premenopausal women.

**Table 1:** Clinico-pathologic characteristics and clinical outcomes of patients treated GnRH agonist treatment.

Authors	Published	Age	Location	Size (cm)	Complete resection		ER status	PR status	GnRHa	Treatment dose	Treatment duration	f/u
Elsaqa et al^[22]^	2022	40	Urethra	7 × 3	NA	+		NA	NA	Monthly	NA	No recurrence in 6 months
Goyal et al^[23]^	2022	40	Lt. labia majora	8 × 8	NA	+		+	Leuprolide	3.75 mg/m		No recurrence in 12 months
Kooy et al^[24]^	2021	39	Vulvar	11 × 9.8 × 2.1	Positive margin	+		NA	Goserelin acetate	NA	18 m	Persistent dz (f/u 66 months)
		49	Vulvar	16 × 7.5 × 5.5	Positive margin	+		NA	Leuprolide acetate	NA	18 m	No recurrence in 26 months
Akhavan et al^[25]^	2021	33	Retroperitoneal (behind vaginal wall)	9.5 × 12	Negative margin	+		NA	NA	NA	6 m	No recurrence in 6 months
Zamani et al^[26]^	2021	28	Vulva	20 × 15 × 10	Complete			NA	Decapeptide	NA	NA	NA
Akram et al^[27]^	2021	premenopausal	Lt. buttock	6.64 × 5.4 × 11.6	Complete	+		+	Leuprolide		Lifelong	No recurrence in 2 months
Walczak et al^[16]^	2021	39	Retroperitoneum	11.3 × 6.5 × 13.0	Incomplete	+		NA	GnRH (LHRH)	3.6 mg/m	6 m	Partial regression
Alomary et al^[28]^	2020	42	Perineum	20 × 15	Incomplete	+		+	Leuprolide	22.5 mg/3 m		No interval change of residual tumor 3 years
Xu et al^[29]^	2020	25	Rt. vulva	5.3 × 5.1 × 4.2		+		+	Triptorelin	3.75 mg		No recurrence in 9 months
Lin et al^[30]^	2019	37	Vestibular gland	3 × 3.3	Positive margin	+		+	NA		6 cycle	No recurrence in 3 years
		55	Rt. chest wall	NA	NA	+		+	NA			
		55	Lt. iliac retroperitoneum	NA	NA	+		+	NA			
Fucà et al^[13]^	2019	46	Perineum	NA		+		+	Triptorelin			PFS 10.3 months
		36	Pelvis	NA		+		+	Triptorelin			PFS 22.5 months
		48	Pelvis	NA		+		+	Triptorelin			PFS 4.8 months
		20	Pelvis	NA		+		+	Triptorelin			PFS 3.6 months
		36	Pelvis	NA		NA		NA	Triptorelin			PFS 6.2 months
		47	Pelvis	NA		+		NA	Triptorelin			PFS 27.5 months
		53	Pelvis	NA		+		+	Triptorelin			PFS 26.4 months
		45	Pelvis	NA		+		+	Leuprorelin			PFS 39.7 months
		43	Perineum	NA		+		-	Triptorelin			PFS 87.2 months
		37	Pelvis	NA		NA		NA	Triptorelin			PFS 79.5 months
		48	Pelvis	NA		+		+	Triptorelin			PFS 7.3 months
Aguilar-Frasco et al^[31]^	2018	39	Vulvar (Rt. labia majora)	28.1 × 10.4	Positive margin	+		+	Leuprolide	3.75 mg/m	NA	No recurrence in 2 years
Song et al^[32]^	2017	49	Vulva (Lt. labia majora), bladder	Positive margin	+	+		Goserelin acetate	NA	NA		No recurrence in 15 months
Im et al^[20]^	2015	22	Rt. vulva	8.9 × 7.3	NA	NA		NA	NA	3.75 mg/m		No recurrence in 10 months
Schwartz et al^[33]^	2014	32	Lt. labia/ischiorectal fossa			+		+	Leuprolide	3.75 mg/m	3 m	PFS 60 months
Gay et al^[34]^	2013	41	Rectovaginal septum	11	NA	+		+	Leuprorelin		6 m	No recurrence in 18 months
Lee et al^[35]^	2011	38	Lt. vulva	6 × 6 × 4	NA	-		+	NA	NA	NA	No recurrence in 20 months
Palomba et al^[17]^	2011	32				+		+	NA	3.75 mg/m	NA	No recurrence in 2 years
Damodaran et al^[36]^	2017	62	Penis, scrotum		Incomplete	NA			NA	3.75 mg/m	20 m	
Mathur et al^[37]^	2015	30	Perineum	6 × 4	NA	+		+	Goserelin	NA	NA	NA

NA = not applicable, PFS = progression free survival.

SERMs have been used as a neoadjuvant therapy to reduce tumor size^[32]^ and as an adjuvant therapy in the recent literature (Table 2).^[15,17–19,37–40]^ SERMs can have antagonistic, agonistic, and neutral effects on specific organs, allowing them to be used in the prevention and treatment of breast cancer and osteoporosis.^[41]^ Tamoxifen, a SERM, has an agonistic effect on the endometrium, whereas raloxifene has an antagonistic effect. Ospemifene, which exerts estrogenic effects on the vaginal epithelium, is used to treat vulvovaginal atrophy and genitourinary symptoms.^[42]^ Since SERMs have diverse effects on different organs, their use in the pelvic cavity and uterus has been reported.^[15,38,39]^ The selective use of SERMs is recommended, especially outside common AAM locations, such as the perineum. Furthermore, AIs have been used as antiestrogen therapies, with reports of maintaining complete regression up to 2 years.^[18,19]^ Similar to GnRH agonists, SERMs and AIs cause side effects, such as arthralgia and vasomotor symptoms, although these have not been documented in previous reports. Further studies are required to develop treatment algorithms based on patient characteristics.

**Table 2:** Clinico-pathologic characteristics and clinical outcomes of patients treated SERM, and AI.

Author	Year	Age	Location	Size (cm)	Complete resection	ER	PR (+)	SERM/AI	Dose	Duration	f/u
Peterknecht et al^[19]^	2021	65	Ischioanal fossa	6.4 × 5.5 × 9.4	Incomplete	+	+	Letrozole	NA	NA	NA
Srivastava et al^[38]^	2021	37				+	+	Tamoxifen	20 mg/day	NA	No recurrence in 2 years
Alosaimi et al^[15]^	2020	35	Abd mass	25.3 × 20 × 8.6		+	+	Raloxifene	NA		No recurrence in 1 year
Husso et al^[18]^	2017	61	Lt. adnexa/ cardiac	6 × 8	NA	+	+	Letrozole	NA		Residual tumor (iliac v, MRI) in 18 months
Sozutek et al^[39]^	2016	35		24 × 12 × 6		+	+	Tamoxifen	20 mg/day	6 m	No recurrence in 2 years
Abu et al^[40]^	2005	46				+	+	Raloxifene	NA	NA	NA
Palomba et al^[17]^	2011	32				+	+	Raloxifene	180 mg/day	NA	No recurrence in 2 years
Mathur et al^[37]^	2015	30 (M)	Perineum	6 × 4	NA	+	+	Goserelin + tamoxifen	NA	NA	NA

ER = estrogen receptor, NA = not applicable, PR = progesterone receptor.

While adjuvant treatment has been proposed, administering the treatment during pregnancy is a significant challenge. The positive ER and PR characteristics of AAM suggest that worsening and recurrence of the disease are possible in women of reproductive age or during pregnancy. However, our understanding of this potential risk is limited due to the scarcity of reports on the disease course. The MITO Rare Tumors Group has recently published a comprehensive review of clinical findings, pathologic characteristics, outcomes, and management of 19 pregnant and 19 nonpregnant patients with AAM of the lower genital tract. This significant research has significantly advanced our understanding of AAM.^[43,44]^ Many more reports are needed to understand the clinical outcomes and management of pregnant AAM patients.

Previous studies have reported no statistical correlation between complete resection and relapse.^[13]^ Similar to previous studies, this study found no significant differences in relapse rates between the complete resection and positive resection margin groups in the recent (Table 3).^[11,38,45–54]^ The follow-up duration was short, with the longest observation period being 31 months. Since AAM has low short-term recurrence rates, longer follow-up periods are necessary to confirm long-term outcomes. Despite the difficulties in predicting relapse or confirming significant predictors, incomplete resection without gross residual tumors resulted in increased tumor size, suggesting that complete resection should be prioritized.^[16,51]^ Staged radical surgery can achieve negative resection margins even in challenging cases.^[11,45]^ Despite positive resection margins, no relapse was observed for 31 months in patients without gross residual tumors.^[54]^ If adjuvant therapy is not feasible postoperatively, staged operations may be planned for a more complete resection.

**Table 3:** Clinico-pathologic characteristics and clinical outcomes of patients treated surgery.

Author	Year	Age	Location	Size (cm)	Complete resection	ER	PR	f/u
Gulino et al^[45]^	2023	46	Lt. labia	10	NA	+	+	
Navitski et al^[11]^	2023	28	Rt. vulva	6.3 × 2.8	RM (+)	+	NA	No recurrence in 8 months
Espejo-Reina et al^[46]^	2022	36	Vesicovaginal space	7 × 3 × 3.5		+	+	NA
Ayati et al^[47]^	2022	31	Rt. labia majora	13.7 × 6.0 × 19.0	Complete	NA	NA	No recurrence in 12 months
York et al^[48]^	2022	31	Vagina	3.5 × 2 × 1.5	Complete, RM (NA)	NA	NA	NA
Djusad et al^[49]^	2021	31	Vagina	5 × 2 × 4		+	+	No recurrence in 1 month
Raptin et al^[50]^	2019	24	Vagina (rectovaginal septum)	7 cm	Complete, RM (NA)	+	+	Recurred in 1 year
Srivastava et al^[38]^	2015	13		7.5 × 5 × 3	RM (+)	+	-	
Zizi-Sermpetzoglou et al^[51]^	2015	47	Rt. labia majora	26 × 21 × 6		NA	+	No recurrence until 18 months/recurred
Abu Saadeh et al^[52]^	2015	34	Cervix	12 × 11 × 10	Complete	NA	NA	No recurrence in 6 months
Choi et al^[53]^	2015	49	Lt. vulva	19 × 19 cm (CT)		ER (+)	NA	No recurrence in 4 years
		31	Retroperitoneum	18 × 15 × 8 cm (CT)	NA	NA	NA	No recurrence in 1 year
		36	Lt. buttock-retroperitoneum	15 × 10 × 6 cm (MRI)	Incomplete	NA	NA	No recurrence in 4 months
Ki et al^[54]^	2014	46	Suprapubic	3.0 × 1.2	NA	NA	NA	No recurrence in 29 months
		18	Lt. labia minora	NA	RM (+)	NA	NA	No recurrence in 31 months

ER = estrogen receptor, NA = not applicable, PR = progesterone receptor, RM = resection margin.

Recent report has suggested chemical ablation with alcohol as a potential new adjuvant therapy, resulting in tumor size reduction and complete remission without side effects.^[55]^

Due to the rarity of the tumor, there was not much research to know whether additional treatment after surgery was necessary and, if so, which treatment would be effective in which patient group, making it difficult to determine treatment priorities. Our table from a recent AAM-related study was collected to determine whether relapse rates differed depending on treatment. However, the lack of long-term outcomes and detailed methods of therapies underscores the need for further research and detailed case reports. These are essential for developing practical treatment algorithms and improving patient outcomes.

## 4. Conclusion

AAM, an exceptionally rare benign mesenchymal tumor, poses a significant diagnostic challenge. Its confirmation requires IHC staining and pathological examination. Due to its high local recurrence rate, long-term follow-up and appropriate adjuvant treatment are crucial to minimize recurrence. However, the scarcity of long-term outcome studies and optimal adjuvant therapies underscores the need for further research and detailed case reports. These are essential for developing practical treatment algorithms and improving patient outcomes.

## Acknowledgments

We would like to thank Professor Kyeong-Min Kim for the pathological analysis.

## Author contributions

**Formal analysis:** Bo Ram Yu, Won Ku Choi.

**Supervision:** Dong Hyu Cho.

**Writing – original draft:** Bo Ram Yu, Won Ku Choi.

**Writing – review & editing:** Dong Hyu Cho, Na-Ri Lee.
